# PHGDH Inhibitor CBR-5884 Inhibits Epithelial Ovarian Cancer Progression via ROS/Wnt/*β*-Catenin Pathway and Plays a Synergistic Role with PARP Inhibitor Olaparib

**DOI:** 10.1155/2022/9029544

**Published:** 2022-09-05

**Authors:** Xiaocui Zhang, Meige Sun, Yisheng Jiao, Bei Lin, Qing Yang

**Affiliations:** Department of Obstetrics and Gynecology, Shengjing Hospital of China Medical University, Shenyang, Liaoning 110004, China

## Abstract

PHGDH attaches importance to serine biosynthesis in cancer cells and maintaining mitochondrial redox homeostasis. However, the role of PHGDH inhibitor CBR-5884 in cell ROS level and its downstream pathways has not been explored in epithelial ovarian cancer. Thus, we investigated the function and possible mechanism of PHGDH inhibitor CBR-5884 on epithelial ovarian cancer in vitro and in vivo. A2780, OVCAR3, and ES-2 were treated with CBR-5884 at different concentrations or different time points. Results showed that CBR-5884 inhibited epithelial ovarian cancer cell proliferation, migration, and invasion and increases cell ROS level. Meanwhile, CBR-5884 exerts antitumor effect through activating ROS/Wnt/*β*-catenin pathway. Besides, CBR-5884 exerts antitumor effect in vivo. What's more, we explored the effect of CBR-5884 with or without PARP inhibitor Olaparib, which showed that the two together had a larger effect. In conclusion, PHGDH inhibitor CBR-5884 inhibits epithelial ovarian cancer proliferation, migration, and invasion through activating ROS/Wnt/*β*-catenin pathway and plays a synergistic role with PARP inhibitor olaparib, which provided a theoretical basis for PHGDH inhibitor CBR-5884 in clinical treatment.

## 1. Introduction

As reported, there were 313,959 cases of newly diagnosed ovarian cancer and 207,252 new deaths for OC in 2020 [1]. EOC is the most common type of ovarian cancer, difficult to find and diagnose in the early stage of disease, deficient in choices of treatment, and easy to relapse [2–5]. Therefore, future exploring the mechanism underlying EOC initiation and progression for further exploring biomarkers to diagnose, treat, and predict the prognosis of EOC is of significance to women in the world.

Serine is a raw material to participate in the synthesis of lipids, proteins and nucleotides and an important source of one carbon unit in cells, which is closely related to the metabolic remodeling of tumor cells [6–8]. As the key enzyme in serine biosynthesis pathway, PHGDH attaches importance to maintaining mitochondrial redox homeostasis, maintaining mitochondrial redox homeostasis [9–12]. Mitochondrial serine produces NADPH and GSH reduction equivalents under SHMT2 catalysis, inhibiting the production of ROS [10, 13, 14]. ROS can also promote antitumor signal transduction and initiate oxidative stress-induced tumor cell death, and one of the antitumor mechanisms of cisplatin is to induce apoptosis by increasing the production of ROS in tumor cells [15–17]. In our recent study, we reported that PHGDH is upregulated at translational level and implicated in platin-resistant in epithelial ovarian cancer cells [18]. However, the role of PHGDH in cell ROS level and its downstream pathways has not been explored in epithelial ovarian cancer. CBR-5884 is one kind of PHGDH inhibitor that have been reported and played a tumor suppressing role in breast cancer [19–23].

Reactive oxygen species which are produced by cell metabolic activities attach importance to cell signal transduction and homeostasis and regulate cell proliferation, apoptosis, and differentiation [24–26]. Recent studies suggested that oxidative stress induced by ROS can regulate Wnt/*β*-catenin signaling pathway in colorectal, breast, lung, pancreatic, and liver cancer [24, 27–31]. Therefore, studying the effect of CBR-5884 and NAC in the regulation of Wnt/*β*-catenin pathway is significant.

PARP inhibitors can enhance the efficacy of radiotherapy and chemotherapy with alkylating agents and platinum drugs by inhibiting DNA damage and repair of tumor cells and promoting apoptosis of tumor cells [32]. Olaparib, a kind of PAPR inhibitor, is mainly used to alleviate the maintenance treatment of platinum sensitive adult patients with recurrent epithelial ovarian cancer, fallopian tube cancer, or primary peritoneal cancer after platinum containing chemotherapy has achieved complete remission [33–35]. Therefore, it is of significance to explore the effect of CBR-5884 with or without olaparib.

Here, we cultured epithelial ovarian cancer cell lines and constructed tumor xenografts model in nude mouse to evaluate the effect of CBR-5884 on epithelial ovarian cancer in vitro and in vivo. Meanwhile, we studied the role of CBR-5884 in cell ROS level and its downstream pathway as well as the effect of CBR-5884 with or without PARP inhibitor olaparib, which may give us a novel prospect in mechanism and clinical treatment of epithelial ovarian cancer.

## 2. Materials and Methods

### 2.1. Materials and Cell Culture

The PHGDH inhibitor CBR-5884, the ROS inhibitor N-acetylcysteine, and the PARP inhibitor olaparib were purchased from MedChemExpress. DMSO was used as control group in cell lines. Through literature reviewing, we used 5 mM NAC treated for 4 h to study its effect [36, 37]. The cell lines (A2780, OVCAR3, and ES-2) were purchased from Procell Life Science & Technology Co., Ltd. (Wuhan, China). A2780 and OVCAR3 were cultured with RPMI 1640 medium (Procell, Wuhan, China) containing 10% FBS (Procell, Wuhan, China), while ES-2 was cultured used McCoy's 5A (Procell, Wuhan, China) containing 10% FBS (Procell, Wuhan, China) in incubator. The incubator purchased from PUHE Biotechnology Co., Ltd. (Wuxi, China) was set at 37 °C, 5% CO2, and 1% O2 with enough humidity.

### 2.2. Cell Viability Assay

A total of 5,000 cells/well were added to 96-well plates and incubated in incubator for 24 h. After different treatment measures, 1 *μ*L of CCK-8 test solution (Procell, Wuhan, China) was added to each well and incubated together for 2 h. The microplate reader was used to measure OD450.

### 2.3. Colony Formation Assay

1000 cells were plated into each well of 6-well plates. After cultured with different treatment measures for 15 days, 4% paraformaldehyde was used for fixing and 1% crystal violet for staining. Colonies meaning cell count >50 were counted.

### 2.4. Apoptosis Condition Assay with Flow Cytometry

The cells were collected after different treatment measures and suspended in PBS. Next, the cells were centrifuged at 4 °C and 1200 rpm for 5 min, and the supernatant was discarded. Use 100 *μ*L binding buffer to resuspend the cells, add 5 *μ*L Annexin V-FITC and 5 *μ*L PI solution (Procell, Wuhan, China), incubate them in room temperature for 15 mins, and add the other 400 *μ*L binding buffer to the mix. Keep away from light and use flow cytometry to detect.

### 2.5. Cell Cycle Distribution with Flow Cytometry

The cells were collected after different treatment measures and suspended in PBS. Next, the cells were centrifuged at 4 °C and 1200 rpm for 5 min, and the supernatant was discarded. Use 75% ethanol to resuspend the cells and fix them at 4 °C overnight. The next day, use PBS to wash the cell precipitation 3 times, resuspend the cells with 500 *μ*L PI/RNase mix (PI 100 *μ*L, RNase 400 *μ*L, Solarbio, Beijing, China) and incubate them together at 37 °C for 30 mins. Keep the tube in ice, away from light, and use flow cytometry to detect.

### 2.6. Transwell Assay

8-*μ*m-pore transwell chambers coated with or without Matrigel (BD, San Diego, USA) (Corning, N York, USA) were used for cell invasion and migration detection. Put 700 *μ*l 20%-FBS containing medium into each bottom chamber, and cells in FBS-free medium (1.0 × 10^4^ cells/200 *μ*l without Matrigel and 3.0 × 10^4^ cells/200 *μ*l with Matrigel) were seeded into the upper chamber. After cultured in the incubator for 24 h, suspended cells in the upper chamber were cleaned out, fix the cells attached to the bottom membrane with 4% paraformaldehyde, and stain with crystal violet. The inverted microscope was used to photograph Images at 200× magnification, and the cells were counted using the image J software.

### 2.7. Cell Oxidative Phosphorylation Level Detection

Cell LDH release level, NADPH level, and GSH level were detected using LDH Cytotoxicity Assay Kit, NADP^+^/NADPH Assay Kit with WST-8, and GSH and GSSG Assay Kit (Beyotime, Shanghai, China) according to the instructions. Besides, cell ROS level was detected using Reactive Oxygen Species Assay Kit (Beyotime) by flow cytometry.

### 2.8. Western Blot

RIPA lysate (Beyotime, Shanghai, China) was used to extract the total protein of cells and tumor xenografts according to the instructions. Then, the extracted total protein was separated by 10% SDS/PAGE and transferred to PVDF membrane (Millipore, Temecula, CA, USA). After blocking in 5% evaporated milk for 2 hours, the membranes were incubated with the main antibody at 4 °C overnight. The next day, the membrane was incubated with secondary antibodies at the ratio of 1 : 5000 for 90 minutes. Protein was visualized with enhanced chemiluminescence (Thermo Scientific, Carlsbad, CA, USA). The detail of primary antibodies is in Table 1.

### 2.9. Animal Study

10 female BALB/Ca-nu nude mice (Beijing Huafukang Biosciences, Beijing, China) in 2 months were maintained in specific pathogen-free (SPF) conditions. The most common epithelial ovarian cancers are serous, mucinous, endometrioid, and clear cell carcinoma. The most common is serous ovarian cancer. Besides, OVCAR3 is one of the commonly and typically used human cell lines for xenograft models [38, 39]. Therefore, 2∗10^7^ OVCAR3 cells were suspended in 150 *μ*L PBS and injected subcutaneously into the axilla of mice. Five days after tumor formation, the mice were treated with physiological saline and CBR-5884 (drug concentration 20 mg/kg) by intraperitoneal injection every two days for 10 days. The long and short diameter of tumor were measured every two days, and the tumor volumes are calculated by V = 1/2∗long diameter∗square of short diameter. All mice were euthanized 15 days later. The tumor weight was measured. Institutional Animal Research Committee of China Medical University approved the animal study.

### 2.10. Immunohistochemistry

5-*μ*m-thick paraffin sections were used for immunohistochemistry. Following deparaffinized, antigen repair and sealed off, the sections were incubated with antibody against PHGDH (1 : 200, 14719-1-AP, Proteintech) or Ki67 (1 : 200, 9027 T, CST) at 4 °C overnight. The slides were incubated with biotinylated goat anti-rabbit antibodies for 1.5 h, stained with diaminobenzidine (abs957, Absin Biotechnology Co., Ltd, Beijing, China) and then counterstained with hematoxylin (abs957, Absin Biotechnology Co., Ltd, Beijing, China). The sections were scored according to the percentage of positive staining cells (0 = negative; 1 = 5–25%; 2 = 26–50%; 3 = 51–74%; and 4 = 75–100%) and the intensity of staining (0 = no staining; 1 = slight staining; 2 = moderate staining; and 3 = strong staining). Scores for the percentage and intensity of staining were added.

### 2.11. Statistical Analysis

Statistical analysis was conducted by unpaired Student's *t*-test in GraphPad Prism 9 (La Jolla, CA, USA). Differences were considered to be statistically significant with *p* < 0.05 (∗*p* < 0.05, ∗∗*p* < 0.01, ∗∗∗*p* < 0.001, and ∗∗∗∗*p* < 0.0001).

## 3. Results

### 3.1. CBR-5884 Inhibits EOC Proliferation, Migration and Invasion

**Figure 1:**
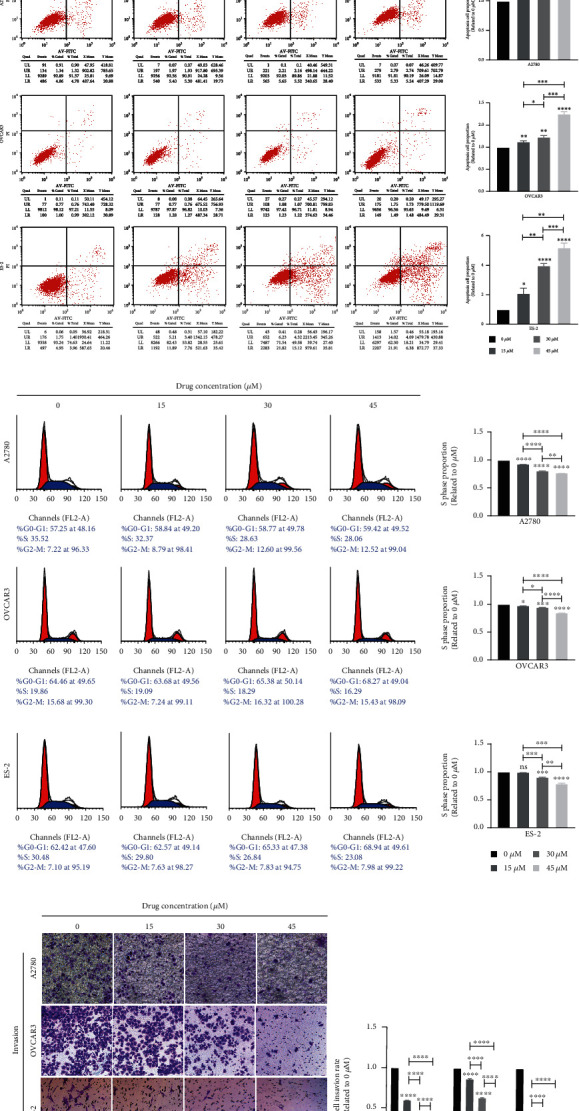
CBR-5884 inhibits EOC proliferation, migration and invasion. (a) Cell viability, (b) colony formation, (c) cell apoptosis, (d) cell cycle, (e) cell migration, and (f) Cell invasion condition of A2780, OVCAR3, and ES-2 cells after treatment of different concentration of CBR-5884.

We conducted our experiments using adenocarcinoma A2780, OVCAR3, and adding clear cell carcinoma ES-2, which involved a relatively comprehensive pathological type of epithelial ovarian cancer cell lines. Besides, we downloaded transcriptomic data of 47 ovary cell lines and listed the PHGDH expression level in Supplementary Table 1 and found that the PHGDH expression level of A2780, OVCAR3, and ES-2 was relatively high. Therefore, we chose A2780, OVCAR3, and ES-2 cell lines for future experiments. Then, cell viability of cells treated with different concentration (0, 10, 20, 30, 45, and 60 *μ*M) of CBR-5884 at different time points (12 h, 24 h, and 48 h) was tested (Figure 1(a)). Accordingly, to keep the inhibition rate, CBR-5884 concentration at 0, 15, 30, and 45 *μ*M was used for other experiments in the form of an isochronous sequence. Besides, OVCAR3, A2780, and ES-2 were treated with CBR-5884 for 24 h, 48 h, and 48 h, respectively. The cell proliferation decreased (Figure 1(b)), apoptosis increased (Figure 1(c)), DNA replication attenuated (Figure 1(d)), cell invasion, and migration weakened (Figure 1(e) and 1(f)) after treatment of 0, 15, 30, and 45 *μ*M CBR-5884. In addition, CBR-5884 inhibits EOC proliferation, migration, and invasion in a dose- and time-dependent manner.

### 3.2. CBR-5884 Increases Cell ROS Level in Epithelial Ovarian Cancer Cell Lines

**Figure 2:**
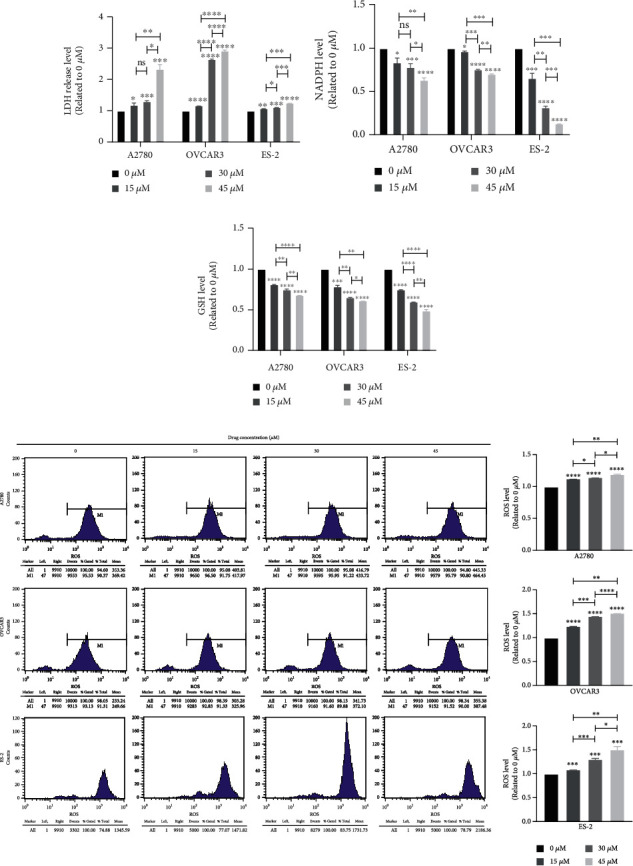
CBR-5884 increases cell ROS level in epithelial ovarian cancer cell lines. (a) LDH release, (b) NADPH, (c) GSH, and (d) ROS level of A2780, OVCAR3, and ES-2 cells after treatment of different concentration of CBR-5884.

After treatment of 0, 15, 30, and 45 *μ*M CBR-5884, the cell LDH release increased (Figure 2(a)), NADPH level reduced (Figure 2(b)), GSH level descended (Figure 2(c)), and ROS level heightened (Figure 2(d)).

### 3.3. CBR-5884 Exerts Tumor Suppression Effect through Increasing Cell ROS Level

To explore whether CBR-5884 inhibits EOC proliferation, migration, and invasion through increasing cell ROS level, we set four group (control group, 5 mM NAC for 4 h, 30 *μ*M CBR for 24 h in OVCAR3, 48 h in A2780 and 48 h in ES-2, and 30 *μ*M CBR for 24 h in OVCAR3, 48 h in A2780, and 48 h in ES-2 with 5 mM NAC for 4 h). NAC is an ROS inhibitor.

**Figure 3:**
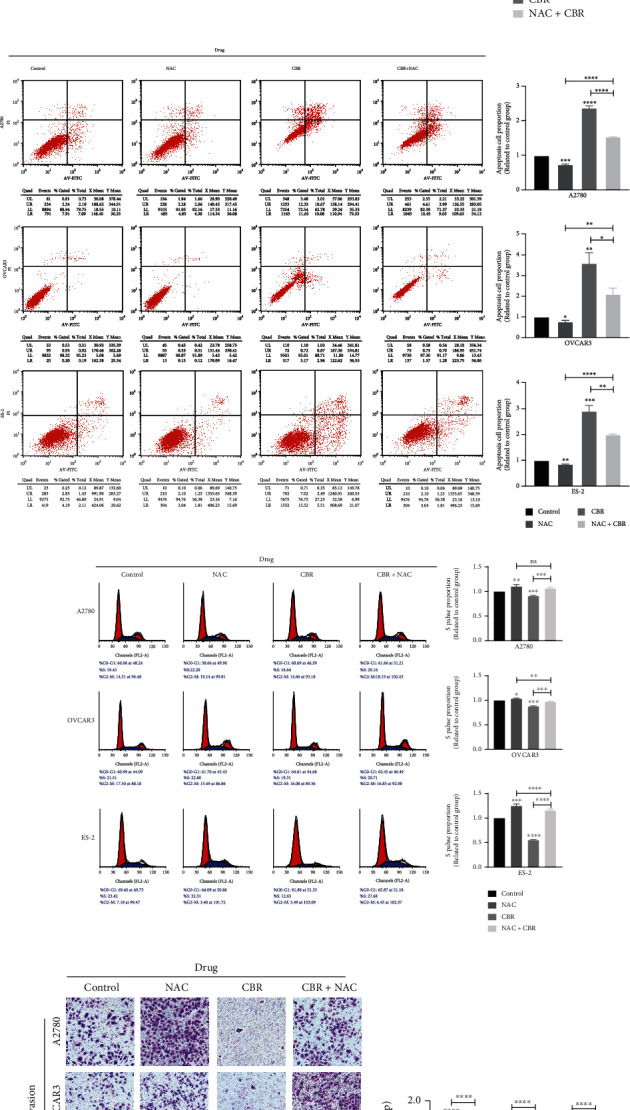
CBR-5884 inhibits EOC proliferation, migration and invasion through increasing cell ROS level. (a) Cell viability, (b) colony formation, (c) Cell apoptosis, (d) cell cycle, (e) cell migration, and (f) cell invasion condition of A2780, OVCAR3, and ES-2 cells after treatment of CBR-5884 with or without NAC.

As shown in Figure 3, NAC promoted EOC malignant biological behavior, CBR-5884 inhibited EOC malignant biological behavior consistent with that in Figure 1, and CBR-5884 together with NAC abolished the tumor suppression effect of CBR-5884.

**Figure 4:**
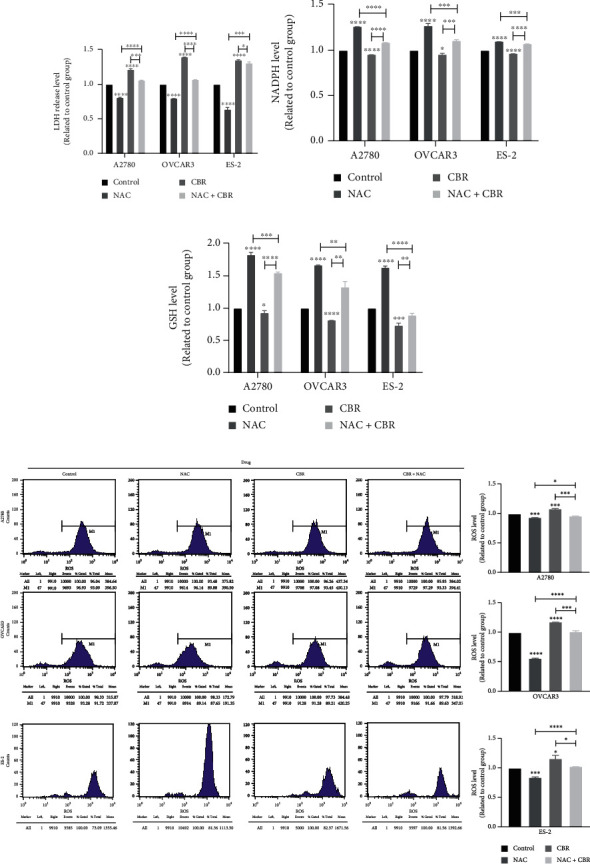
NAC rescues the effect of CBR-5884 in epithelial ovarian cancer cell lines. (a) LDH release, (b) NADPH, (c) GSH, and (d) ROS level of A2780, OVCAR3, and ES-2 cells after treatment of CBR-5884 with or without NAC.

Additionally, NAC decreased the cell LDH release level (Figure 4(a)), heightened NADPH level (Figure 4(b)), increased GSH level (Figure 4(c)), and reduced ROS level (Figure 4(d)). CBR-5884 promoted the ROS level consistent with that in Figure 2. What's more, CBR-5884 together with NAC abolished the ROS promoting effect of CBR-5884 (Figures 4(a)–4(d)).

### 3.4. CBR-5884 Exerts anti-Tumor Effect through Activating ROS/Wnt/*β*-Catenin Pathway

**Figure 5:**
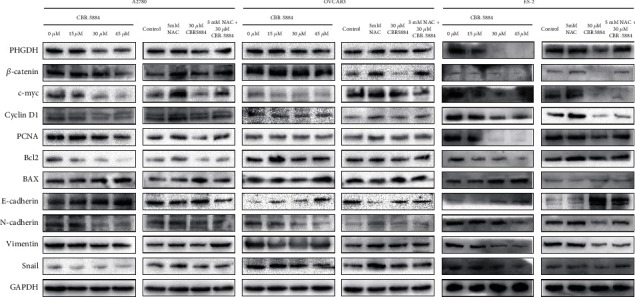
CBR-5884 exerts antitumor effect through activating ROS/Wnt/*β*-catenin pathway. PHGDH, *β*-catenin, c-myc, Cyclin D1, PCNA, Bcl2, BAX, E-caderin, N-cadherin, vimentin, and Snail expression level after treatment of different concentration of CBR-5884 or CBR-5884 with or without NAC.

To future understand the mechanism in drug inhibition of the epithelial ovarian cancer, we detected the downstream molecules changes. As shown in Figure 5, after using 0, 15, 30, and 45 *μ*M CBR-5884, PHGDH and pathway-related indicators *β*-catenin, c-myc, cyclin D1, PCNA, Bcl2, N-cadherin, vimentin, and Snail expression level declined increasingly, and BAX and E-cadherin ascended increasingly. Besides, NAC promoted expression of PHGDH and pathway related indicators *β*-catenin, c-myc, cyclin D1, PCNA, Bcl2, N-cadherin, vimentin, and Snail and inhibited expression of BAX and E-cadherin. Meanwhile, CBR-5884 together with NAC abolished the inhibiting effect of CBR-5884 on PHGDH and pathway-related indicators *β*-catenin, c-myc, cyclin D1, PCNA, Bcl2, N-cadherin, vimentin, Snail, and the promoting effect of CBR-5884 on BAX and E-cadherin (Figure 5).

### 3.5. CBR-5884 Exerts Antitumor Effect In Vivo

**Figure 6:**
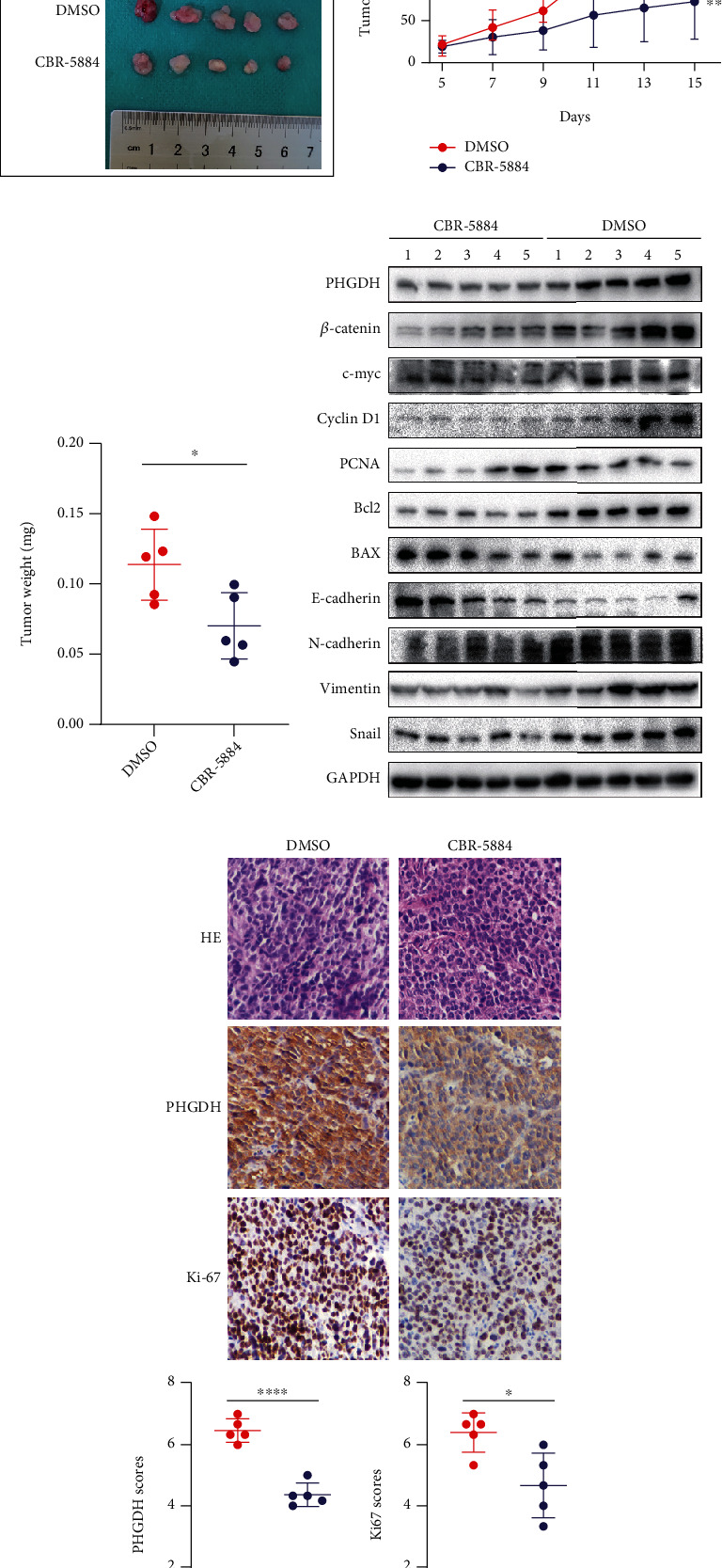
CBR-5884 exerts anti-tumor effect in vivo. (a) Tumor xenograft, (b) tumor volume, (c) tumor weight, (d) related protein level via WB, and (e) HE staining, PHGDH, and Ki67 expression level via IHC in two groups.

To explore the tumor suppression effect of CBR-5884 in vivo, we used mice xenograft model. Tumor grew more slowly and tumor weight was lighter in group of intraperitoneal injection of CBR-5884 (Figure 6(a)). We also examined the protein level of tumor tissues in two groups, which showed that PHGDH and pathway-related indicators *β*-catenin, c-myc, cyclin D1, PCNA, Bcl2, N-cadherin, vimentin, and Snail expression level were lower and BAX and E-cadherin higher in CBR-5884 group (Figure 6(d)). The immunohistochemical staining of tumor tissues between two groups showed that PHGDH and Ki67 expressions were inhibited in CBR-5884 group (Figure 6(e)).

### 3.6. CBR-5884 Plays a Synergistic Role with Olaparib

**Figure 7:**
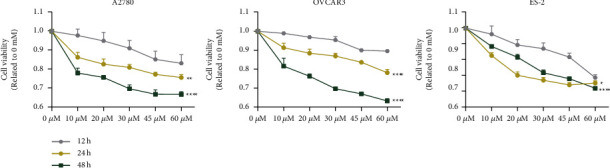
Olaparib inhibits EOC viability. Cell viability condition of A2780, OVCAR3, and ES-2 cells after treatment of different concentration of olaparib.

**Figure 8:**
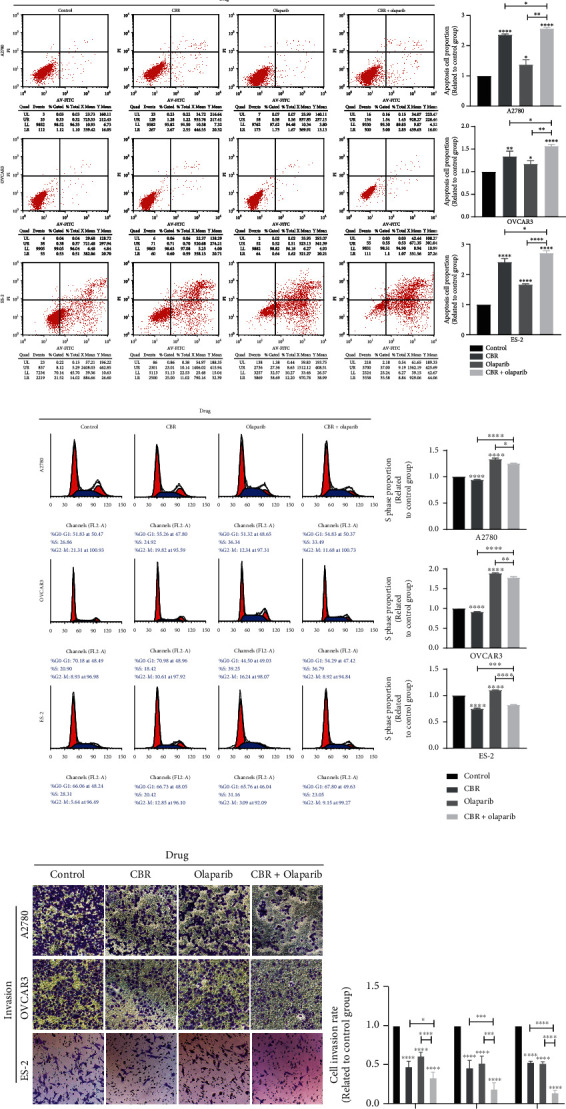
CBR-5884 and olaparib synergistically inhibits the progression of EOC. (a) Cell viability, (b) colony formation, (c) cell apoptosis, (d) cell cycle, (e) cell migration, and (f) cell invasion condition of A2780, OVCAR3, and ES-2 cells after treatment of CBR-5884 with or without olaparib.

First, cell viability of cells treated with different concentration (0, 10, 20, 30, 45, and 60 *μ*M) of olaparib at different time points (12 h, 24 h, and 48 h) was tested (Figure 7). As shown in Figure 7, cell viability of OVCAR3, A2780, and ES-2 treated with olaparib for 24 h were approximately 60%-70% inhibited under 60 *μ*M. As shown in Figure 1, cell viability of OVCAR3, A2780, and ES-2 treated with CBR-5884 for 24 h was approximately 60%-70%, 30%-40%, and 60%-70% inhibited under 60 *μ*M, respectively. According to the inhibition rate of OVCAR3, we set CBR and olaparib in a 1 : 2 concentration ratio (control group, 10 *μ*M olaparib and 20 *μ*M CBR, 20 *μ*M olaparib and 40 *μ*M CBR, and 30 *μ*M olaparib and 60 *μ*M CBR for 24 h in A2780, OVCAR3, and ES-2) to explore the effect of CBR-5884 with or without PARP inhibitor olaparib. And we found that CBR-5884 played a synergistic role in cell viability (Figure 8(a)), proliferation (Figure 8(b)), apoptosis (Figure 8(c)), invasion (Figure 8(e)), and migration (Figure 8(f)) with PARP inhibitor olaparib. However, after using olaparib, cell S phase proportion was increased, and co-effect of CBR-5884 and olaparib abolished the effect of using alone (Figure 8(d)).

## 4. Discussion

Since EOC causes so many deaths in the world every year, and many patients are suffering from this kind of excruciating disease, it deserves more in-depth exploration and research. Metabolic reprogramming is a mechanism by which cells promote cell proliferation and growth by changing metabolic mode in order to meet energy needs and can be an important marker cell of malignant tumor [40, 41]. Serine synthesis increases in cancer cells and is the third largest metabolic-related substance of cancer cells after glucose and glutamate [6, 8]. PHGDH is the first key enzyme of serine anabolism, which is increased in some tumors and is closely related to poor prognosis [42–44]. Importantly, silencing PHGDH gene can significantly affect the growth of PHGDH-dependent cancer, making the enzyme a new target for cancer treatment [10, 11, 14]. In our recent study, we found that PHGDH was upregulated in epithelial ovarian cancer and was regulated by lncRNA RMRP and DDX3X in translational level [18]. However, how PHGDH affects its downstream pathways remains a question and has not been explored in EOC.

Considering clinical practicability, we used PHGDH inhibitor CBR-5884 to conduct this study. First, we explored the role of CBR-5884 in EOC malignant biological behavior. The results showed that CBR-5884 exerts an antitumor effect. Next, we explored the effect of CBR-5884 on epithelial ovarian cancer cell ROS level. It is reported that cell LDH level, NADPH level, and GSH level could reflect the cell oxidative stress condition and affect cell ROS level [45, 46], so we studied the level of LDH, NADPH, GSH, and ROS. The result showed that CBR-5884 can increase the ROS level, which is consistent with the results that have been reported [22, 47–50]. Thus, we speculated that CBR-5884 exerts an antitumor effect through inducing ROS imbalance in cells. We added N-acetylcysteine (NAC) to observe whether adding NAC could abolish the tumor suppression effect of CBR-5884. NAC is an ROS inhibitor. If CBR-5884 exerts tumor suppression effect through increasing cell ROS level, then adding NAC could abolish the tumor suppression effect of CBR-5884. As expected, we confirmed that CBR-5884-mediated ROS imbalance was necessary in the tumor suppression effect and that ROS level and CBR-5884 were indeed related and CBR-5884 was highly likely to play a role by affecting intracellular ROS levels. However, there is an interesting phenomenon; when treated with NAC+ CBR, the GSH level in ES-2 cell line is lower than control when it is expected that the GSH level will be higher, which may be because the GSH level in ES-2 cell line is different and specific, which brought us a new perspective to our future research.

Next, we examined the expression level of *β*-catenin and the pathway-related genes, such as c-myc and Cyclin D1. We also tested the proliferation and apoptosis-related genes, such as BAX, Bcl2, and PCNA. Meanwhile, we tested the EMT-related genes, such as E-cadherin, N-cadherin, vimentin, and Snail. The results showed that CBR-5884 exerts an antitumor effect through activating ROS/Wnt/*β*-catenin pathway, which is consistent with the reported results in other malignant tumors [24, 27–31]. At last, we verified the effect of CBR-5884 in vivo through nude mice xenograft. The results were consistent with that in vitro. Besides, tumor tissues in CBR-5884 group had lower expression of Ki67, the prognostic indicator commonly used in clinical.

PARP inhibitors represented by olaparib can be used not only for first-line maintenance treatment and second-line maintenance treatment, but also for post-line rescue treatment, “all online,” with significant curative effect. The studies confirmed that maintenance therapy represented by PARP inhibitors has changed the traditional treatment of epithelial ovarian cancer. Therefore, we explored the effect of CBR-5884 with or without PARP inhibitor olaparib, which showed that the two together had larger effect in cancer inhibition, which gave us a novel idea to clinical therapy. However, after using olaparib, cell S phase proportion was increased, and co-effect of CBR-5884 and olaparib abolished the effect of using alone. Thus, the underlying mechanism of CBR-5884 and olaparib together needs to be further studied. While the safety and effectiveness of combined use also need being tested by clinical trials.

Through our research, we can get a knowledge of the tumor suppressing role of PHGDH inhibitor CBR-5884 in epithelial ovarian cancer via regulating the ROS level and the downstream Wnt/*β*-catenin signaling pathway. What's more, we concluded that CBR-5884 played a synergistic role with PARP inhibitor olaparib. In the future, we will promote the exploration about molecular targeted therapy based on PHGDH to prevent or reverse epithelial ovarian cancer and ovarian cancer cisplatin resistance in clinical.

## 5. Conclusion

In conclusion, our study demonstrated that PHGDH inhibitor CBR-5884 inhibits epithelial ovarian cancer proliferation, migration, and invasion through activating ROS/Wnt/*β*-catenin pathway and plays a synergistic role with PARP inhibitor olaparib. These provided a theoretical basis for PHGDH inhibitor CBR-5884 in clinical treatment.

## Figures and Tables

**Table 1:** The detail of primary antibodies.

	Company	Lot no.	Dilution ratio
PHGDH	Proteintech	14719-1-AP	1 : 1000
*β*-Catenin	Wanleibio	WL0962a	1 : 1000
c-myc	Proteintech	10828-1-AP	1 : 1000
Cyclin D1	Wanleibio	WL01435a	1 : 1000
PCNA	Proteintech	10205-2-AP	1 : 1000
Bcl2	Proteintech	12789-1-AP	1 : 1000
BAX	Proteintech	50599-2-Ig	1 : 1000
E-cadherin	Proteintech	20874-1-AP	1 : 5000
N-cadherin	Proteintech	22018-1-AP	1 : 2000
Vimentin	Proteintech	10366-1-AP	1 : 2000
Snail	Wanleibio	WL01863	1 : 1000
GAPDH	Proteintech	10494-1-AP	1 : 5000

## Data Availability

The results data used to support the findings of this study are included within the article.

## Abbreviations

OC: Ovarian cancer
EOC: Epithelial ovarian cancer
PHGDH: Phosphoglycerate dehydrogenase
ROS: Reactive oxygen species
NAC: N-acetylcysteine
CCK-8: Cell counting kit-8
FBS: Fetal bovine serum
OD450: Optical density at 450 nm
PI: Propidium iodide
LDH: Lactate dehydrogenase
GSH: Glutathione
SPF: Specific pathogen-free
IHC: Immunohistochemistry
DAB: Diaminobenzidine.

## Supplementary Materials

Supplementary Materials Supplementary Table 1. PHGDH expression level in different cell lines from CCLE database.
