# Supplementary material for: PHGDH Inhibitor CBR-5884 Inhibits Epithelial Ovarian Cancer Progression via ROS/Wnt/β-Catenin Pathway and Plays a Synergistic Role with PARP Inhibitor Olaparib
Source: Oxid Med Cell Longev. 2022 Sep 5;2022:9029544. doi: 10.1155/2022/9029544 (PMC9467758; doi:10.1155/2022/9029544)
Supplement: Supplementary Materials — Supplementary Table 1. PHGDH expression level in different cell lines from CCLE database. [file 9029544.f1.docx]

**Supplementary table 1 PHGDH expression level in different cell lines from CCLE database**

| ID | PHGDH |
| --- | --- |
| OVTOKO_OVARY | 1.00E+00 |
| SKOV3_OVARY | 5.13E+00 |
| COV434_OVARY | 6.02E+00 |
| OV7_OVARY | 6.42E+00 |
| JHOC5_OVARY | 1.06E+01 |
| JHOM1_OVARY | 1.27E+01 |
| CAOV4_OVARY | 1.28E+01 |
| OAW42_OVARY | 1.29E+01 |
| JHOM2B_OVARY | 1.34E+01 |
| IGROV1_OVARY | 1.42E+01 |
| OVMANA_OVARY | 1.44E+01 |
| EFO27_OVARY | 1.58E+01 |
| RMUGS_OVARY | 1.88E+01 |
| OVKATE_OVARY | 1.91E+01 |
| RMGI_OVARY | 2.01E+01 |
| TOV21G_OVARY | 2.21E+01 |
| JHOS2_OVARY | 2.37E+01 |
| COV644_OVARY | 2.55E+01 |
| OV56_OVARY | 2.60E+01 |
| SNU840_OVARY | 2.82E+01 |
| SNU8_OVARY | 2.87E+01 |
| TYKNU_OVARY | 3.04E+01 |
| COV318_OVARY | 3.20E+01 |
| MCAS_OVARY | 3.32E+01 |
| KURAMOCHI_OVARY | 3.46E+01 |
| OVSAHO_OVARY | 3.55E+01 |
| OELE_OVARY | 3.58E+01 |
| OVK18_OVARY | 3.63E+01 |
| OC314_OVARY | 3.71E+01 |
| FUOV1_OVARY | 3.89E+01 |
| SNU119_OVARY | 3.93E+01 |
| OVCAR8_OVARY | 3.99E+01 |
| COV362_OVARY | 4.07E+01 |
| TOV112D_OVARY | 4.33E+01 |
| OVISE_OVARY | 4.40E+01 |
| OAW28_OVARY | 5.08E+01 |
| EFO21_OVARY | 5.43E+01 |
| **ES2_OVARY** | **5.68E+01** |
| ONCODG1_OVARY | 6.10E+01 |
| **NIHOVCAR3_OVARY** | **6.13E+01** |
| HEYA8_OVARY | 6.32E+01 |
| CAOV3_OVARY | 6.41E+01 |
| 59M_OVARY | 7.96E+01 |
| JHOS4_OVARY | 8.47E+01 |
| OVCAR4_OVARY | 8.54E+01 |
| **A2780_OVARY** | **1.04E+02** |
| OV90_OVARY | 1.32E+02 |
